# Prooxidant effects of high dose ascorbic acid administration on biochemical, haematological and histological changes in *Cavia porcellus* (Guinea pigs): a Guinea pig experimental model

**DOI:** 10.11604/pamj.2023.46.18.36098

**Published:** 2023-09-13

**Authors:** Oladunni Omolabake Otanwa, Uche Samuel Ndidi, Abdulrazak Baba Ibrahim, Emmanuel Oluwadare Balogun, Kola Matthew Anigo

**Affiliations:** 1 Department of Biochemistry, Faculty of Life Sciences, Ahmadu Bello University, Zaria, Kaduna State, Nigeria,; 2 Department of Biochemistry, Faculty of Science, University of Uyo, Uyo, Akwa Ibom State, Nigeria

**Keywords:** Ascorbic acid, urea, creatinine, serum electrolytes, inflammation, histopathology, haematological, antioxidant

## Abstract

**Introduction:**

Ascorbic acid (AA) is a water-soluble vitamin that is well known for its antioxidant and immune-boosting properties. Owing to the wide-range application of AA in the treatment of numerous ailments and its sweet taste, it is usually abused i.e. overused. However, the effect of the abuse has rarely received attention. Therefore, this study was designed to assess the effect of oral administration of high-dose ascorbic acid on biochemical and haematological parameters as well as the effects on the kidney, liver and lungs.

**Methods:**

adult guinea pigs were divided into four (4) groups where group 1 served as the untreated control group and groups 2-4 were dosed with 29 mg, 662 mg and 1258 mg of ascorbic acid per day, respectively for 28 days.

**Results:**

the result revealed that administration of high dose ascorbic acid significantly (P<0.05) increased serum creatinine from 50.0 ± 7.09 (NC) to AA29- 73.8 ± 4.5, AA-662-89.7 ± 3.3 and AA1258- 79.9 ± 5.7mmol/L and urea levels in the treatment group AA-1258 -18.3 ± 0.5 µmol/L compared to the normal group (NC-2.15 ± 0.6 µmol/L). Disturbance in electrolyte balance was observed with a significant (P<0.05) increase in Na+ from NC- 131.3 ± 3.5 mmol/L to 135.7 ± 3.6 mmol/L in the AA-1258 treatment group, Cl- ( NC- 67.1 ± 1.6 mmol/L increased to AA29- 92.1 ± 0.83, AA662- 95.3 ± 1.3 and AA-1258- 95.6 ± 0.4 mmol/L), and Ca2+ (NC- 2.66 ± 0.03 to AA1258- 3.36 ± 0.03 mmol/L) and a significant (P<0.05) decrease in serum K+ in the AA29-5.0 ± 0.2, AA662-5.2 ± 0.3 and AA1258-5.6 ± 0.3 mmol/L treatment groups compared to the normal group 6.6 ± 0.3 mmol/L. There was also a significant (P<0.05) increase in the differential blood count in the animals with a significant (P<0.05) increase in red blood count ( NC-5.11 ± 0.13 ×10^6^/µL to AA1258- 5.75 ± 0.11×10^6^/µL ), haematocrit count (NC 39.90 ± 0.52% to AA-29-42.08 ± 0.24 and AA1258-46.13 ± 0.86%), white blood count (NC 10.15 ± 1.01 ×10^3^/µL to AA1258- 15.18 ± 1.65×10^3^/µL ), total lymphocytes (NC 3.5 ± 0.51×10^3^/µL to AA29-5.28 ±0.43×10^3^/µL), monocytes (NC 0.45 ± 0.07×10^3^/µL to AA1258 0.80 ± 0.07×10^3^/µL), eosinophils (NC 0.23 ± 0.03×10^3^/µL to AA12580.40 ± 0.03×10^3^/µL), basophils (NC0.68 ± 0.10×10^3^/µL to AA12581.20 ± 0.10×10^3^/µL) and neutrophil count (NC 4.73 ± 0.68×10^3^/µL to AA1258 8.36 ± 0.71×10^3^/µL). The histopathological indices indicate cellular necrosis in the AA662 and AA1258 treatment groups of the kidney and liver respectively compared to the normal control which has normal cells.

**Conclusion:**

high dose of ascorbic acid can therefore be suggested to cause damage to the cells by causing cellular necrosis as observed in the histopathology results and has effect on the blood cells as observed in the increase compared to the normal control, and the consequences are possibly triggered through inflammatory responses.

## Introduction

Ascorbic acid (AA) is a water-soluble vitamin that is often consumed by humans as a supplement. It is known for its strong reducing property and free radical scavenging activity [1,2]. Furthermore, AA is useful for increasing iron absorption and is a therapeutic adjuvant in iron chelation therapy [3]. Therapeutically, it is used in the treatment of common cold, sepsis, advanced cancer, and burns [4-6]. Although AA is often prescribed for its immune-boosting and antioxidant properties, reports abound on its ability to switch from its physiological antioxidant role to a prooxidant role usually at high concentrations leading to pathological conditions. The prooxidant effect of AA and its potential detrimental side effects are often neglected [7].

Ascorbic acid is synthesized from D-glucoronate in both plants and animals through a series of reactions with L-gulonolactone oxidase catalyzing the final step in the biosynthetic pathway. It is synthesized in the kidneys of fish, amphibians, and reptiles, and the liver of some mammals [8]. This vitamin is mainly obtained by humans through the consumption of fruits and vegetables such as oranges, lemons, guavas, tomatoes, and pepper. It could be used as a food additive and a dietary supplement [9].

Upon ingestion, ascorbic acid is partially converted into oxalate and excreted in the urine thus, increasing the potential risk of calcium oxalate formation when taken in excess due to the composition of the end products of ascorbic acid degradation [10]. This degradation involves the hydrolysis of dehydroascorbate to 2,3-diketo-L-gulonate and is spontaneously degraded to oxalate, CO_2_ and L-erythrulose [11]. The deficiency of vitamin C results in scurvy with clinical presentation of haemorrhage, hyperkeratosis and haematological abnormalities [12]. Adverse effects of ascorbate over-supplementation include headaches, nausea, vomiting, diarrhoea, abdominal cramps, insomnia, flushing and dizziness in addition to increased risk of kidney stones and elevation of uric acid and oxalate due to its acidification effect in urine [13]. Sustained levels of ascorbic acid could be toxic and immunosuppressive for human T cells [14]. As a result, prolonged administration of high-dose ascorbic acid could lead to systemic oxalosis with clinical presentation of kidney injury [15]. Therefore, the purpose of this study is to investigate the effect of long-term administration of a high dose of AA on the biochemical, haematological, and histopathological indices in guinea pigs, which will provide an insight into the likely pattern in humans.

## Methods

**Study design and setting:** the study was a randomized control trial comparing between 3 intervention groups (AA29, AA662, AA1258) and control group. Like humans, guinea pigs are incapable of AA synthesis hence the choice of guinea pigs as model animals for this study. The animals were maintained under standard environmental conditions (25 ± 2°C, 45 ± 10% relative humidity, light-dark cycle of 12 h) in the animal house belonging to the Department of Veterinary Pathology, Ahmadu Bello University, Zaria- Nigeria, and were given access to a standard pellet diet (Vital Feeds, Jos, Nigeria) and water *ad libitum*. The study was conducted in Kaduna State, Nigeria between June - August 2020.

**Participants:** twenty-four (24) healthy adult guinea pigs weighing between 250 - 500 g were obtained from the Faculty of Veterinary Medicine animal house in Ahmadu Bello University, Kaduna State, Nigeria. The inclusion criterion was the state of health after the period of acclimatization, which was two weeks.

**Experimental study groups and treatment:** animals were further divided randomly into four treatment groups of six animals each. Each treatment group comprised six (6) animals, which were treated in the following manner: ***Group I***- animals were given only 1 mL distilled water -Negative Control (NC). ***Group II***- animals treated with ascorbic acid (29 mg/day) (AA29). ***Group III***- animals treated with ascorbic acid (662 mg/day) (AA662). ***Group IV***- animals treated with ascorbic acid (1258 mg/day) (AA1258). L-ascorbic acid was purchased from Sigma-Aldrich® scientific company (Darmstadt, Germany). Administration of the supplements was via the oral intubation for 28 days. On the last day, we sacrificed the animals under chloroform anaesthesia and a part of the blood collected in 5 mL EDTA bottles, while another part collected in plain bottles for further analysis including serum urea, creatinine and electrolytes. We collected blood in plain bottles and allowed it to clot for 2 h at room temperature (25-26°C) before centrifuging using a KJLC-1 centrifuge (Kangjian, Jiangsu, China) for 15 minutes at 1000 × g. The top layer was collected as a serum for assay and stored at -20°C.

**Haematological assays:** assays for packed cell volume (PCV), haemoglobin concentration (Hb), red blood cell (RBC), leucocytes and platelet counts, and mean corpuscular volume (MCV) were carried out using the automated analyser (SYSMEX K2XIN, Shangai, China), which operated on the principle of the cyanomethaemoglobin and light intensity for the differential leucocytes count [16].

### Biochemical analyses

***Creatinine:*** Jaffe's picric acid method was used for the determination of serum creatinine as described by [17]. The method was based on the reaction of creatinine with alkaline picrate to develop a red-coloured complex. The colour produced from the sample was compared in a colourimeter at 500 nm with the colour produced by a known amount of creatinine under the same condition. The intensity of the colour formed was proportional to the creatinine concentration in the sample. Serum urea was determined using the Urease-Berthelot method as described by [18]. Briefly, exactly 100 µL of sample and standard was added to 1000 µL of working reagent in clean test tubes, while 100 µL of distilled water was added to the blank. The absorbance of the sample and standard were read against that of the blank at 500 nm, after 30 seconds and 90 seconds.

### Calculation


$$\text{Creatinine }\left(\mu \text{mol}/L\right)=\frac{\Delta \text{Absorbance of sample}\times \text{Concentration of standard}}{\Delta \text{Absorbance of standard}}$$

***Urea:*** the serum urea was determined using the Urease-Berthelot method as described by Doumas *et al*. [18]. This was based on the hydrolysis of urea into ammonia ion and carbondioxide in the presence of urease as a catalyst. Ammonia ion reacted with Berthelot's reagent to form a blue coloured complex in a reaction known as Berthelot's reaction. Berthelot's reagent is an alkaline solution of phenolic chromogen and hypochlorite. The intensity of the blue colour formed was proportional to the urea content which was calculated colorimetrically at 546 nm. Briefly, 10 µL sample was added to 1000 µL of working reagent in clean test tubes, while 10 µL of distilled water was added to the blank. The resulting mixture was incubated for 5 minutes at 37°C before reading the absorbance. The absorbance of the sample and standard were read against that of the blank at 546 nm.

### Calculation


$$\text{Urea }\left(\text{mmol}/L\right)=\frac{\text{Absorbance of sample}\times \text{Standard Concentration}}{\text{Absorbance of Standard}}$$

***Determination of serum electrolytes:*** the method used for the determination of serum electrolytes involved the use of the ion-selective electrodes as described by Bakker and Qin [19]. The electrolytes determined include sodium, potassium, chloride and calcium ions.

**Histopathological examination:** the histo pathological examination was carried out at the histology laboratory of the Department of Human Anatomy, Faculty of Medical Sciences, Ahmadu Bello University, Nigeria. The examination was carried out by the method of Culling [20]. Briefly, guinea pigs' kidneys and lungs were fixed in 10% buffered formalin. The sections were processed and trimmed for paraffin sectioning in a paraffin tissue-processing machine. Sections of the kidney and lungs were made to a thickness of about 5 µm and these tissues were stained with haematoxylin and eosin for microscopic examination. The sections were viewed at a magnification of × 400.

**Ethical statement:** ethical approval was obtained from Ahmadu Bello University, Zaria, Ethical Committee on Animal Use and Care (ABUCAUC). All animals used in this study were handled based on ethical guidelines on the use of animals for research purposes as stipulated by ABUCAUC.

**Statistical analyses:** results in this study were expressed as mean ± standard deviation (mean ± SD) using the Statistical Package for Social Sciences (SPSS) version 20, Chicago, Illinois, USA. One-way analysis of variance (ANOVA) was used to analyze the data obtained with differences in means compared using Duncan Multiple Range *posthoc* test. Values of P < 0.05 were considered significant.

## Results

### Biochemical analyses

**Figure 1:**
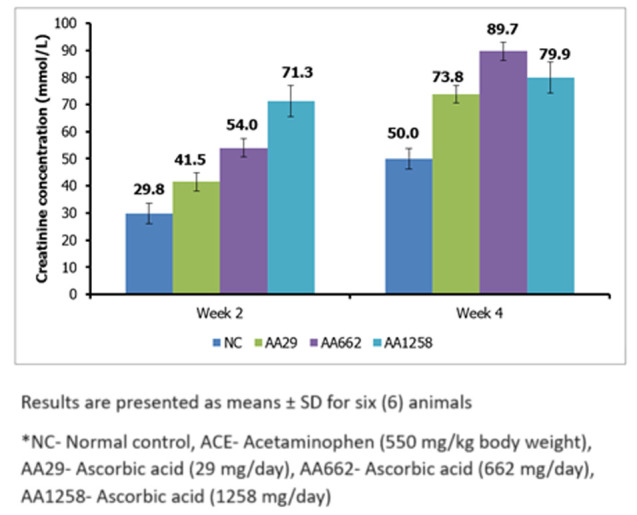
mean serum creatinine concentration of Guinea pigs treated with ascorbic acid

***Creatinine:*** serum creatinine analyses indicate that after 2 weeks of AA administration, there were significant (P< 0.05) increases in serum creatinine. The creatinine level in the AA1258 treatment group was 71.3 ± 8.9 mmol/L while it was just 29.8 ± 3.8 mmol/L in the control group, as shown in Figure 1. After the fourth week, a general increase in creatinine levels across the groups was noted compared to the control group, however, the increases amongst the treated groups were not significant (P< 0.05). The values in the AA29, AA662 and AA1258 were 73.8 ± 4.5 mmol/L, 89.7 ± 3.3 mmol/L and 79.9 ± 5.7 mmol/L respectively (Figure 1).

**Figure 2:**
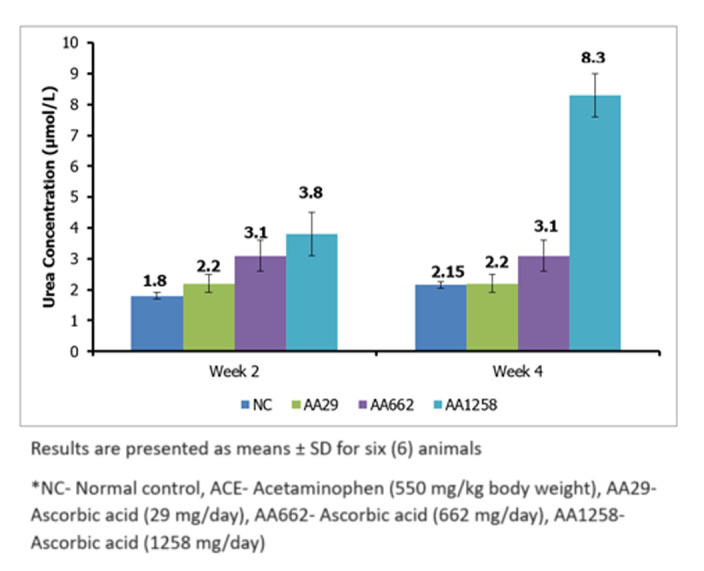
serum urea concentration of Guinea pigs treated with ascorbic acid

***Urea:*** level of serum urea followed the same pattern as creatinine. Serum urea level increased significantly after administration of different doses of AA (Figure 2). After 2 weeks of administration, the serum urea concentration in the AA662 groups was 3.1 ± 0.5µmol/L and 3.8 ± 0.7 µmol/L respectively, as compared to 1.8 ± 0.1 µmol/L observed in the control group. Four (4) weeks after, serum urea in the AA1258 group increased dramatically up to 8.3 ± 0.5µmol/L, while it remained almost unchanged in the control group (Figure 2).

**Figure 3:**
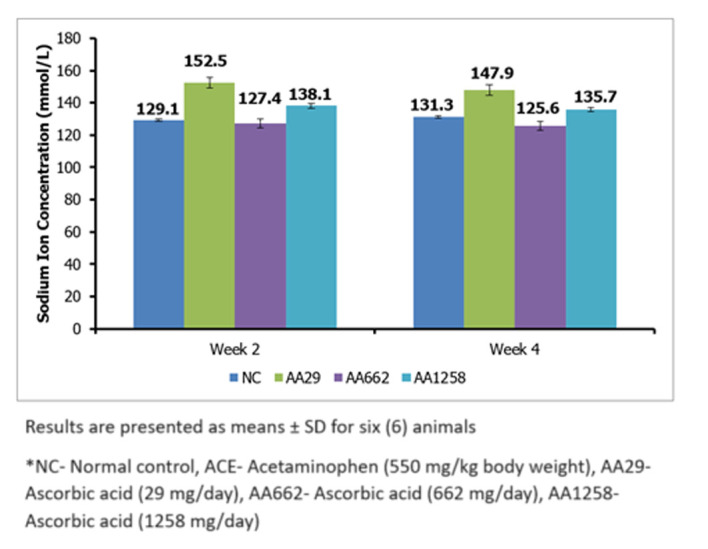
serum sodium concentration of Guinea pigs treated with ascorbic acid

**Figure 4:**
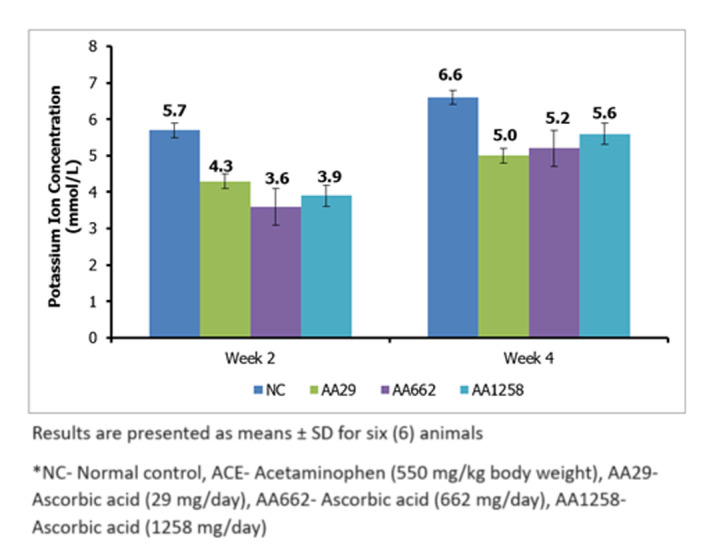
serum potassium concentration of Guinea pigs treated with ascorbic acid

***Serum electrolytes*** the average concentration for serum sodium ion in the various groups after the first two weeks of AA administration, significantly (P < 0.05) increased from 129.1 ± 0.8 mmol/L in the NC group to 152.5 ± 3.2 mmol/L and 138.1 ± 1.3 mmol/L in the AA29 and AA1258 treatment groups respectively (Figure 3). After 4 weeks of administration of AA, the concentration of serum sodium ion remained approximately at the same level in the normal control and the treated groups (Figure 3). Concentration of serum potassium ion showed a significant (P < 0.05) decrease across the treatment groups versus the normal control group. The level in the control was 5.7 ± 0.2 mmol/L, and after 2 weeks of administration of AA, potassium ion concentration dropped to 3.5 - 4.3 mmol/L (Figure 4).

**Figure 5:**
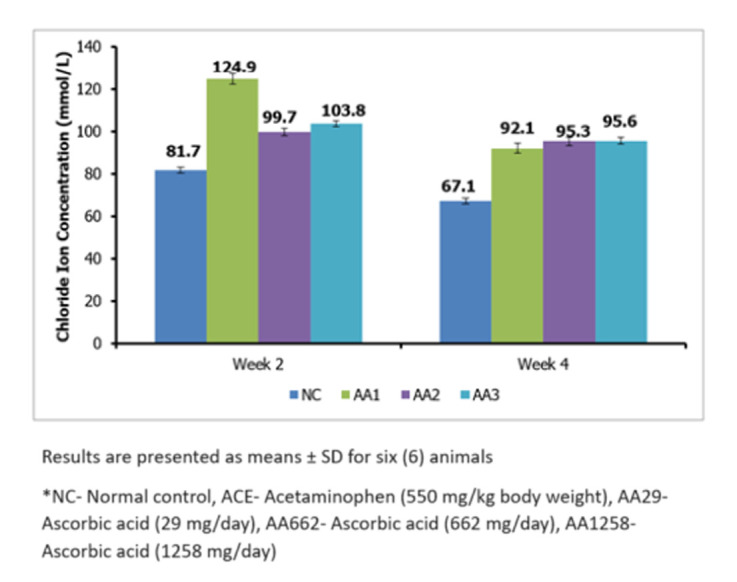
serum chloride concentration of Guinea pigs treated with ascorbic acid

**Figure 6:**
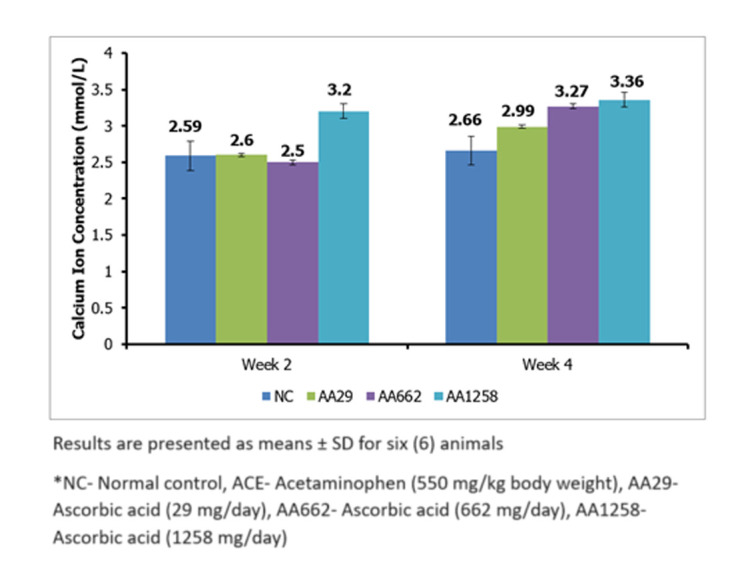
serum calcium concentration of Guinea pigs administered with ascorbic acid

Serum Chloride ion concentration increased significantly (P < 0.05) in the AA29, AA662 and AA1258 treatment groups with values 124.9 ± 2.4 mmol/L, 99.7 ± 1.8 mmol/L and 10^3^.8 ± 1.5 mmol/L respectively in reference to the control group which was 81.7 ± 1.4 mmol/L, after 2 weeks of treatment (Figure 5). After four weeks of AA administration, the values remained significantly higher for the treatment groups than the control group. The concentration of serum calcium ion significantly (P < 0.05) increased in the AA1258 treatment group with a value of 3.2 ± 0.1 mmol/L about the control group, which had a value of 2.59 ± 0.2 mmol/L (Figure 6). However, at the end of the fourth week, all the treatment groups had significantly increased (P < 0.05) with AA29, AA662 and AA1258 having values of 2.99 ± 0.02 mmol/L, 3.27 ± 0.02 mmol/L and 3.36 ± 0.03 mmol/L while the control group remained relatively the same.

**Haematology assays:** there are significant (P < 0.05) increases in erythrocyte indices in the AA-administered animals (Table 1). Estimated red blood cell counts for animals in the NC group were 5.11 ± 0.13 (10^6^/µL ) whereas it was 5.75 ± 0.11(10^6^/µL ) for the AA-1258 group (Table 1), and correspondingly, the haematocrit values were 39.90 ± 0.52% and 46.13 ± 0.86%, respectively. Likewise, the total haemoglobin significantly (P < 0.05) increased for the AA-1258 group (15.83 ± 0.43 g/dL) compared to all the other groups including the NC (14.25 ± 0.17 g/dL). On the contrary, there was no significant change across the groups for MCV, MCH and MCHC values when compared to the NC group (Table 1).

**Table 1:** effects of high-dose ascorbic acid administration on erythrocyte indices

Erythrocyte Indices	NC	AA-29	AA-662	AA-1258
RBC (×10^6^/µL)	5.11 ±0.13^a^	5.35 ± 0.06^a^	5.31 ±0.07a	5.75 ±0.11^b^
HGB (g/dL)	14.25 ±0.17^a^	14.88 ±0.14^a^	14.20 ±0.15^a^	15.83 ±0.43^b^
HCT (%)	39.90 ±0.52^a^	42.08 ±0.24^b^	40.90 ±0.46^ab^	46.13 ±0.86^c^
MCV (fL)	77.98 ±0.96^ab^	78.50±1.02^ab^	77.14±1.06^a^	79.13±1.43^ab^
MCH (pg)	27.22 ±0.29^a^	27.02 ±0.47^a^	27.58 ±0.78^a^	27.17 ±0.25^a^
MCHC (g/dL)	34.93 ±0.43^as^	34.44 ±0.52^a^	35.70 ±0.68^a^	34.35 ±0.61^a^
PLT (×10^3^/μL)	435.25 ±5.76^a^	535.33 ±11.02^b^	553.00 ±41.55^b^	542.30 ±41.55^b^

Results are presented as means ±SD; Values with different superscripts across the row are significantly different from each other at P < 0.05. *AA- Ascorbic acid, HCT- Haematocrit count, HGB- Haemoglobin concentration, MCH- Mean Cell Haemoglobin, MCHC- Mean cell haemoglobin concentration, MCV- Mean cell volume, RBC- Red blood count, PLT-Platelets. AA29- Ascorbic acid 29 mg/day, AA662-Ascorbic acid 662 mg/day, AA1258- Ascorbic acid 1258 mg/day, NC- Normal Control

The white blood cell count showed a significant (P < 0.05) increase in the AA1258 treatment group with 15.18 ± 1.65 (10^3^/µL compared to the control group 10.15 ± 1.01(10^3^/µL as shown in Table 2. Similarly, the lymphocyte count showed a significant (P < 0.05) increase in the AA29 and AA662 groups having values of 5.28 ± 0.43 and 4.36 ± 0.40 (10^3^/µ) respectively compared to the control with 3.50 ± 0.51(10^3^/µL. There was a significant (P < 0.05) increase in the AA1258 group for the neutrophil, Basophil, eosinophil and monocyte counts which were 8.36 ± 0.71, 1.20 ± 0.10, 0.40 ± 0.03 and 0.80 ± 0.07 (10^3^/µL respectively compared to the control group. Neutrophil lymphocyte ratio (NLR) indicated significant (P < 0.05) increase in the AA1258 group with ratio (3.13 ± 0.33) when compared to the control group and AA29 groups (1.43 ± 0.27 and 1.41 ± 0.21 respectively).

**Table 2:** effects of high-dose ascorbic acid on leucocyte indices

Leucocyte Indices	NC	AA29	AA662	AA1258
WBC (×10^3^/μL)	10.15 ± 1.01^a^	9.84 ± 0.48^a^	13.15 ± 1.15^ab^	15.18 ± 1.65^b^
LYMP (×10^3^/μL)	3.50 ± 0.51^ab^	5.28 ± 0.43^c^	4.36 ± 0.40^bc^	2.46 ± 0.23^a^
NEUT (×10^3^/μL)	4.73 ± 0.68^a^	5.12 ± 0.25^a^	6.27 ± 0.39^a^	8.36 ± 0.71^b^
BASO (×10^3^/μL)	0.68 ± 0.10^a^	0.74 ± 0.04^a^	0.91 ± 0.06^a^	1.20 ± 0.10^b^
EOSIN (×10^3^/μL)	0.23 ± 0.03^a^	0.25 ± 0.01^a^	0.30 ± 0.18^a^	0.40 ± 0.03^b^
MONO(×10^3^/μL)	0.45 ± 0.07^a^	0.49 ± 0.02^a^	0.60 ± 0.04^a^	0.80 ± 0.07^b^
NLR	1.43 ± 0.27^a^	1.41 ± 0.21^a^	2.20 ± 0.49^ab^	3.13 ± 0.33^b^

Results are presented as means ± SD; Values with different superscripts across the row are significantly different from each other at P < 0.05. *AA- Ascorbic acid, BASO- Basophil count, EOSIN- Eosinophil count, LYMP- Lymphocyte count, MONO- Monocyte count, NEUT- Neutrophil count, NLR- Neutrophil lymphocyte ratio WBC- White blood cell count. AA29- Ascorbic acid 29 mg/day, AA662-Ascorbic acid 662 mg/day, AA1258- Ascorbic acid 1258 mg/day, NC- Normal Control

**Figure 7:**
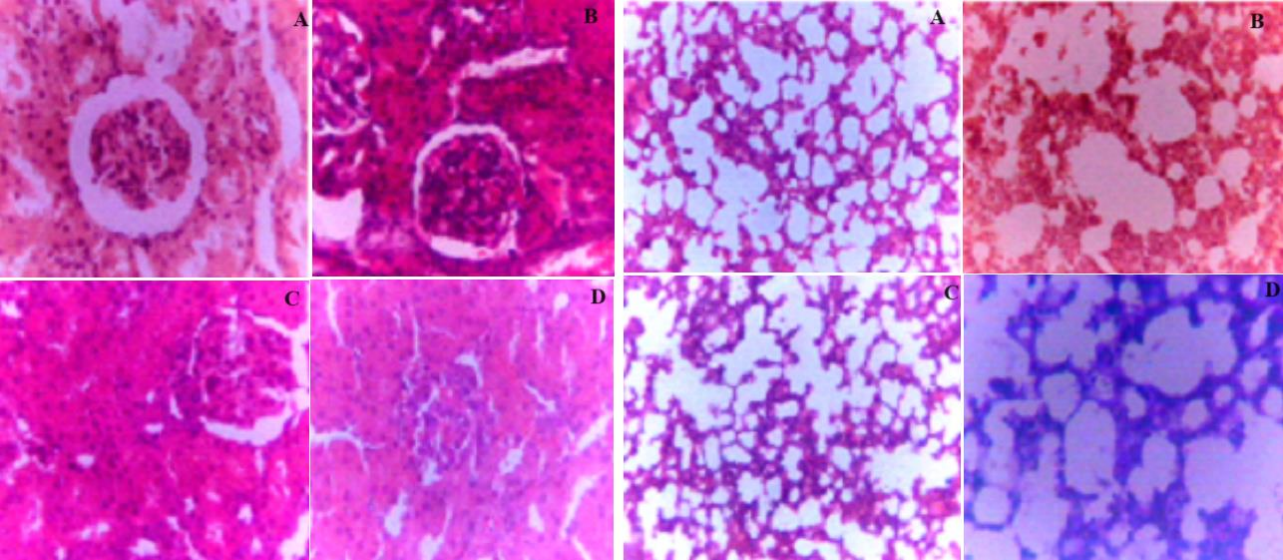
panel 1: photomicrograph of kidneys of Guinea pigs: A) normal control group showing normal kidney tubules and glomerulus; B) ascorbic acid treatment group (29mg/day) showing hyperplasia of inflammatory cells and tubular adhesion; C) ascorbic acid treatment group (662mg/day)showing tubular adhesion, tubular necrosis of cells and hyperplasia of inflammatory cells; D) group treated with high-dose ascorbic acid (1258mg/day) showing severe tubular adhesion, tubular necrosis of cells and hyperplasia of inflammatory cells; panel 2: photon micrograph of the lungs of Guinea Pigs: A) normal control group showing normal alveoli; B) ascorbic acid treatment group (29mg/day) showing alveoli congestion; C) ascorbic acid treatment group (662mg/day) showing hyperplasia of inflammatory cells; D) group treated with high-dose ascorbic acid (1258mg/day) showing hyperplasia of inflammatory cells, hematoxylin and Eosin stain, magnification x100

**Histopathology:** histological observation of the effect of high-dose ascorbic acid on the kidneys is shown in the Figure 7 (panel I). The Guinea pigs had varying degrees of tubular adhesion, hyperplasia of inflammatory cells and tubular necrosis in the treatment groups. Figure 7 (panel II) is a photomicrograph showing the histological architecture of the lungs of the guinea pigs indicating hyperplasia of inflammatory cells.

## Discussion

The chief objective of this research endeavor is to understand the likely patterns that might be seen in humans by studying the effects of prolonged exposure to high doses of ascorbic acid (AA) on guinea pigs, particularly focusing on the changes in their biochemical, hematological, and histopathological markers. Our study has discovered a significant shift in the renal tissue histology, along with an increase in serum creatinine and urea levels that suggest a serious renal failure. As the research continued, we also noticed a shift in the concentration of serum electrolytes: the levels of sodium, chloride, and calcium were elevated while the concentration of potassium ions decreased. The high doses of ascorbic acid administered throughout the study caused a noticeable increase in the neutrophil-lymphocyte ratio and levels of red blood cells (RBCs), hemoglobin (Hb), hematocrit (HCT), white blood cells (WBCs), neutrophils, basophils, and eosinophils.

The gradual rise in the serum creatinine and urea levels during the course of our study is likely related to the enhancement of kidney malfunction or kidney disease, as exemplified in acute kidney injury (AKI) in a study by Kellum *et al*. [21]. When compared with the regular control group, the levels of ascorbic acid treatment groups align with the findings of Maliakel *et al*. [22] who explored cisplatin-induced kidney function toxicity. Elevated urea nitrogen levels may also be attributed to reduced blood flow to the kidneys due to dehydration and blood failure, characterized by platelet dysfunction, coagulopathy, epitheliopathy, and insufficient tissue oxygen supply [23]. Urea, as a primary nitrogenous waste product of protein metabolism, is expelled exclusively via the kidneys, indicating kidney dysfunction.

Our findings of increased sodium, chloride, and calcium levels coincide with the research outcomes of Meng [24] and Biobaku *et al*. [25], in which ascorbic acid caused a rise in these levels. Hypernatremia, hyperchloremia, and hypokalemia conditions likely to occur due to substantial water loss possibly caused by vomiting, diarrhea (characteristic of high-dose ascorbic acid administration), and dehydration [25] were also noted. Hypercalcemia may develop due to renal calcium retention, thereby exacerbating tubular necrosis and triggering AKI as concurrent increase in calcium and creatinine signals kidney function loss [26].

Hematology, the study of blood and blood-forming tissues' morphology, aids in assessing the health status of animals exposed to toxins and stressors, thereby assisting in diagnosing and prognosticating health conditions [27]. Our findings reveal that the administration of exceedingly high-dose ascorbic acid led to an increase in RBC, Hb, HCT, WBC, neutrophils, basophils, eosinophils, and monocytes, ultimately leading to an elevated neutrophil-lymphocyte ratio, an indicator of oxidative stress. The rise in RBC count could be a result of decreased oxygen levels in the blood, potentially leading to increased secretion of erythropoietin, a hormone. Such a scenario could be triggered by conditions like pulmonary fibrosis or kidney diseases. A surge in RBC levels might also prelude kidney stone formation due to the accumulation of uric acids in the form of sodium urate crystals in supersaturated blood [28]. Hb, a heme-carrying protein in RBCs, also witnessed a substantial increase, consistent with Biobaku *et al*.'s research [25] that observed a surge in RBC after high-dose ascorbic acid treatment. The rise in RBC, Hb, and HCT levels might be attributed to dehydration caused by vomiting and diarrhea, early signs of digestive disturbances attributed to high intake of ascorbic acid [29]. This could lead to reduced plasma volume, hence increasing the RBC and hemoglobin concentration, overloading the kidneys, and leading to kidney dysfunction, kidney stones, and gout [30]. A boost in platelet count might be due to enhanced bone marrow platelet production. In group AA1258, the low lymphocyte count and high neutrophil count might indicate an impaired immune system as evidenced by the high neutrophil-lymphocyte ratio (NLR), a sensitive biomarker for diagnosing sepsis and systemic inflammation, both crucial in AKI's pathophysiology [31]. An increase in WBC and neutrophils could signal an inflammatory reaction [32]. The AA1258 group, treated with the highest ascorbic acid dose, displayed a substantial increase in monocytes, basophils, and eosinophils, suggesting the immune system's response to a foreign body or stress, inflammation, infections, blood disorders, or tissue damage [33]. The increase in monocytes aligns with a study associating higher monocyte turnover with lung tissue damage as the disease progressed in AIDS research [34].

The kidney's normal histology displays tubules and glomerulus, part of epithelial tissues containing cells responsible for reabsorbing useful materials into the blood [35]. Tubular adhesion, a reaction to injury, infection, or radiation, triggers an inflammatory response activating immune cells, leading to WBC recirculation and migration [36]. Damage to kidney tubule cells could cause acute tubular necrosis resulting from reduced blood flow, thereby limiting oxygen supply to the kidneys [37]. This chain of events could cause AKI as proposed by [38], with epithelial cell-cell adhesion implicated in renal injury leading to cytokine and other inflammatory mediator release, resulting in leukocyte accumulation at injury sites [39]. Inflammatory hyperplasia, as described by Shukla *et al*. [40], is a benign soft tissue response to a local irritant, leading to inflammatory cell infiltrate comprising lymphocytes and plasma cells. Crystal deposition, infectious processes, progressive nephropathy, or direct chemical administration could lead to renal tubule inflammation [41].

Alveoli, the functional units of the lungs, are tiny air sacs responsible for oxygen uptake. Alveoli congestion, characterized by the alveoli filling up with excess fluid, impedes gaseous exchange, leading to erythrocyte egression in the alveolar spaces [42]. Hypoxic lung inflammation is induced by red blood cells initiating an acute inflammatory response [43]. A connection between AKI and acute lung injury (ALI), a disruption of the normal lung histology leading to high-permeability pulmonary edema due to inflammation or oxidation-mediated injury to the alveoli-capillary barrier and downregulation of the epithelial active-ion transport system, was suggested. Acute respiratory distress may result from blood gas disturbances compromising renal blood flow and leading to AKI [43]. In a study conducted by Sinnberg *et al*. [44] using high-dose AA on cancer cells, an increase in ascorbyl radicals correlated with rising serum concentrations, thereby reducing serum oxygen levels.

This study was performed during the hot-wet season and the animal selection was randomized to eliminate bias. Analyses were conducted using one-way analysis of variance and Duncan's multiple range test was used as the post-hoc test, meaning exact p-values were not obtained as would have been the case with a T-test.

## Conclusion

From the results obtained from this study, prolonged administration of high-dose ascorbic acid causes changes in serum biomarkers of kidney function such as serum creatinine, urea and electrolytes balance which are indicators of kidney injury. The increase in RBC, WBC, neutrophils and lymphocytes is indicative of tissue injury due to the migration of neutrophils and lymphocytes to the site of damage as observed in the renal and pulmonary tissue with necrosis of cells observed. High-dose ascorbic acid thus could be said to affect electrolyte concentration and body fluid balance thus affecting equilibrium of major fluids, electrolytes.

### What is known about this topic

- *Ascorbic acid is an organic compound that occurs readily in nature;*
- *It serves as a potent antioxidant;*
- *High doses of ascorbic acid has been implicated in the treatment of cancers, common colds and burns*.

### What this study adds

- *Evidence of pro oxidant role of ascorbic acid has been indicated by damage to renal and pulmonary cells when used in high-dose;*
- *High-dose ascorbic acid could result increase in haematological parameters such as RBC, neutrophils and lymphocytes;*
- *High-dose ascorbic acid could result in increased renal injury biomarkers like serum creatinine, urea and electrolytes*.
